# Structure and function of a novel lineage-specific neutralizing epitope on H protein of canine distemper virus

**DOI:** 10.3389/fmicb.2022.1088243

**Published:** 2023-01-11

**Authors:** Zhenwei Bi, Wenjie Wang, Xingxia Xia

**Affiliations:** ^1^Institute of Veterinary Medicine, Jiangsu Academy of Agricultural Sciences, Key Laboratory of Veterinary Biological Engineering and Technology, Ministry of Agriculture and Rural Affairs, National Center for Engineering Research of Veterinary Bio-products, Nanjing, China; ^2^Jiangsu Key Laboratory of Zoonosis, Jiangsu Co-innovation Center for Prevention and Control of Important Animal Infectious Diseases and Zoonoses, Yangzhou, China; ^3^College of Veterinary Medicine, Nanjing Agricultural University, Nanjing, China

**Keywords:** canine distemper virus, H protein, epitope, structure, neutralization mechanism

## Abstract

Canine distemper virus (CDV) infects many sensitive species worldwide and its host range is expanding. The hemagglutinin (H) protein, the major neutralizing target, binds to cellular receptors and subsequently triggers fusion for initial viral infection. So it’s necessary to clarify the precise neutralizing epitopes of H protein and extend the knowledge of mechanisms of virus neutralization. In this study, a neutralizing monoclonal antibody (mAb) 2D12 against CDV H protein, which had different reactivity with different CDV strains, was generated and characterized. A series of truncated H proteins were screened to define the minimal linear epitope 238DIEREFD244 recognized by 2D12. Further investigation revealed that the epitope was highly conserved in America-1 vaccine lineage of CDV strains, but different substitutions in the epitope appeared in CDV strains of the other lineages and two substitutions (D238Y and R241G) caused the change of antigenicity. Thus, the epitope represents a novel lineage-specific neutralizing target on H protein of CDV for differentiation of America-1 vaccine lineage and the other lineages of CDV strains. The epitope was identified to localize at the surface of H protein in two different positions in a three-dimensional (3D) structure, but not at the position of the receptor-binding site (RBS), so the mAb 2D12 that recognized the epitope did not inhibit binding of H protein to the receptor. But mAb 2D12 interfered with the H-F interaction for inhibiting membrane fusion, suggesting that the mAb plays key roles for formation of H-F protein oligomeric structure. Our data will contribute to the understanding of the structure, function, and antigenicity of CDV H protein and mechanisms of virus neutralization.

## Introduction

Canine distemper (CD) caused by canine distemper virus (CDV) is a contagious disease with multisystemic infections including respiratory, gastrointestinal, and neurological system. The cross-species infections make CDV become a global multi-host pathogen including non-human primates (Beineke et al., 2009). Therefore, it still faces enormous challenges to control the spread of CDV. As a member of the genus *Morbillivirus* of the family *Paramyxoviridae*, CDV is an enveloped virus and possesses two types of glycoproteins on the viral envelope, hemagglutinin (H) and fusion (F) proteins. CDV H is a type II glycoprotein with 607 amino acids of ∼78 kD, which contained N-terminal cytosolic tail (amino acids [aa] 1 to 35), single-pass transmembrane domain (aa 36 to 58), and ectodomain (aa 59 to 604 or 607). The ectodomain is comprised of three domains: membrane-proximal stalk region (aa 59 to 154), connecting region (aa 155 to 187) and membrane-distal head domain (aa 188 to 604 or 607) with six-sheets arranged in a six-bladed propeller fold (Hashiguchi et al., 2007; Herren et al., 2018). Two head monomers are linked with disulfide bonds at Cys139 and Cys154 to form a homodimer, which is further assembled into a tetrameric structure by forming a dimer of dimers (Herren et al., 2018). CDV H protein binds to cellular receptors on the target host cells, which triggers F protein-mediated membrane fusion between the virus envelope and host cell plasma membrane (von Messling et al., 2001; Avila et al., 2014). But the details involved in these steps are largely unknown. The neutralizing antibodies against CDV H protein may target the receptor-binding site (RBS) or the regions involved in interacting with the F protein and membrane fusion (Tahara et al., 2013). The identification of neutralizing epitopes will extend the knowledge of mechanisms of virus neutralization and infection.

Canine distemper virus vaccines have been widely used for control of CD. Although neutralizing antibodies directed against each of H and F glycoproteins on the surface of the viral envelope are elicited, H protein-specific antibodies mainly account for the protection against CDV infection (Hirayama et al., 1991; Bouche et al., 2002; de Swart et al., 2005, 2009). But the antigenic epitopes of CDV H protein have not been investigated clearly. Based on the high variability of H protein, more and more lineages are recognized for CDV, despite the endemic lineages are vary in different countries or regions (Espinal et al., 2014; Bi et al., 2015a; Anis et al., 2018a; Duque-Valencia et al., 2019; Rendon-Marin et al., 2019; Wang et al., 2022). CDV vaccine strains belong to the American-1 lineage, while circulating CDV strains in susceptible animals with CD belong the other lineages. Nevertheless, several studies have suggested that currently circulating CDV strains show genetic, antigenic variations, which may potentially affect the efficacy of vaccination (Bi et al., 2015b; Anis et al., 2018b). The direct evidence to prove the difference of neutralizing antigenic epitopes of CDV H protein is lacking.

In the present study, we identified a novel neutralizing epitope and its location on the CDV H protein structure, analyzed the conservation and variability of the neutralizing epitope among different lineages of CDV strains. We also identified the neutralizing mechanism of the mAb 2D12 that recognized the epitope. These data will provide potential uses for the development of diagnostic methods and new antiviral strategies, and contribute to our understanding of the antigenicity and membrane fusion mechanism of CDV.

## Materials and methods

### Viruses, cells, and antibodies

An older CDV 851 strain of America-1 lineage (Bi et al., 2017) has been cultured and passaged in Vero cells in our laboratory. Two Chinese field isolates CDV NJ(11)2 of Asia-4 lineage, NJ(12)3 of Asia-1 lineage were isolated using Vero cells expressing dogSLAM (Vero-dogSLAM), and their H genes were characterized in our previous study (Bi et al., 2015a; Bi, 2022). Vero-dogSLAM cells were cultured and grown in DMEM (GIBCO, USA) supplemented with 10% fetal calfserum (FCS) and 0.1 mg/ml of Zeocin (Invitrogen, USA); 293T and Vero cells were cultured and grown in DMEM (GIBCO, USA) supplemented with 10% FCS at 37°C in the presence of 5% CO_2_, respectively. Cells were transfected using Lipofectamine 2000 (Invitrogen, USA). Anti-Flag mouse monoclonal antibody (F1804, Sigma, USA), anti-Myc rabbit polyclonal antibody (R1208-1, Hangzhou Hua An Biotechnology Co., Ltd., Hangzhou, China), anti-SLAM rabbit polyclonal antibody (A2044, ABclonal, Wuhan, China), HRP-anti-mouse goat polyclonal antibody (074-1806, KPL, USA), HRP-anti-rabbit goat polyclonal antibody (074-1506, KPL, USA) were purchased from commercial sources.

### Production of mAb

BALB/c mice were immunized with purified CDV 851 strain to prepare mAb as previously described (Bi et al., 2015b). Briefly, the splenocytes from the mouse were fused with sp2/0 myelomas using polyethylene glycol 4000 (Merck, Germany). The hybridomas were selected by hypoxanthineaminopterin-thymidine medium (HAT) (Sigma, USA) and cloned by limiting dilution. The indirect immunofluorescence assay (IFA) was used to identify specificity of mAbs. Mice ascites were generated from the paraffine primed BALB/c mice injected with the hybridomas, which were purified by HiTrap Protein G Affinity chromatography column. The isotypes of mAbs were determined by mouse antibody isotyping kit (Sigma, USA).

### Indirect ELISA

ELISA plates were coated with the antigen (5 μg/mL) in bicarbonate buffer at 4°C overnight. The plates were washed with PBS containing 0.05% Tween-20 (PBST) and blocked with PBST containing 10% FCS at 37°C for 2 h. After washing, mAb 2D12 or dog serum were added and incubated at 37°C for 1 h. Subsequently, horseradish peroxidase (HRP)-conjugated goat anti-mouse/dog IgG was added and incubated at 37°C for 1 h. After washing, the plates were incubated with substrate solution tetramethyl benzidine (TMB) at 37°C and the reaction was stopped with 2 M H_2_SO_4_. The OD 450 nm was read in an automatic ELISA plate reader.

### Western blot

The protein samples were mixed with 6 × SDS-PAGE Loading Buffer (Beyotime, Shanghai, China), boiled at 90°C for 5 min and separated on 10% SurePAGE Bis-Tris gels (GenScript, Nanjing, China). Separated proteins were then transferred to nitrocellulose membranes (NC), and the membranes were blocked with PBS containing 5% non-fat milk at 4°C overnight. The membranes were washed three times with PBST, then incubated with the various antibodies for 1 h at 37°C. After washing, HRP-conjugated secondary antibody was added and incubated for 1 h at 37°C. The membranes were washed again and the protein bands were detected with enhanced chemiluminescence (ECL) kit (Thermo, USA).

### Immunofluorescence assay

Vero cells infected with CDV 851 strain, Vero-SLAM cells infected with CDV NJ(11)2 strain, and 293T cells transfected with recombinant H protein were washed once with PBS and fixed in the pre-cooling ethanol absolute for 30 min at 4°C and washed with PBST for three times, followed by incubation in mAbs 2D12, G3N or 2B9 for 1 h at 37°C. After washing in PBST, cells were incubated with the fluorescein isothiocyanate (FITC)-conjugated goat anti-mouse IgG (BOSTER, Wuhan, China) for 1 h at 37°C. Finally, the cells were washed and observed under the fluorescence microscopy (Olympus, Japan).

### Virus neutralization assay

Serial 10-fold dilutions of mAb 2D12 in duplicate were prepared in DMEM and then mixed with an equal volume of 200 TCID_50_ of CDV 851 strain. After incubation for 1 h at 37°C, the virus-antibody mixture was inoculated onto Vero cells in 96-well microplates and plates were incubated at 37°C in the presence of 5% CO_2_. The cytopathic effect (CPE) was observed for 5–10 days and antibody titer of virus neutralization (VN) was evaluated by Reed-Muench method.

### Construction of expression plasmids

The H proteins of different CDV strains with FLAG-tag at the N terminal region were cloned into pCAGGS vector. A series of large truncated H proteins were amplified by PCR and inserted into pET28a with *Eco*R I and *Xho* I. To further identify the precise epitope, the reactive polypeptides of H protein were truncated by one residue at their C-terminus and/or N-terminus until the smallest binding domain recognized by mAb were identified. The complementary primer pairs of the oligonucleotides contained *EcoR* I and *Xho* I restriction sites were synthesized, denatured at 95°C for 5 min and annealed at 25°C for 30 min. Then the products were cloned into the same sites of pGEX-4T-1 vector. These recombinant plasmids were transformed into *E. coli* BL21 (DE3) to express each fragment containing GST tag and their reactivity with mAb was tested by western blot.

### Alignment and location of CDV H epitope

To investigate the conservation of the epitope to CDV, a total of 405 representative CDV strains from different lineages were selected from the GenBank database, which were listed in Supplementary Table 1. The amino acids sequence of the identified epitope was aligned among CDV strains of different lineages using DNAstar MegAlign software. The homology of CDV H protein was modeled by SWISS-MODEL online server, and the localization of neutralizing epitope on H protein was analyzed by PyMOL software.

### The binding of virus to cells

CDV 851 and mAb 2D12 were mixed evenly and treated at 37°C for 1 h, then inoculated into monolayer Vero cells or 293T/SLAM cells at 4°C for 2 h. After washing with pre-cooling PBS for 3 times, and the cells were collected and total RNA was extracted using TRIzol (Vazyme, Nanjing, China). Equal concentrations of RNA (1 μg) were transcribed to cDNA using a ReverAid First Strand cDNA Synthesis Kit (Thermo, USA) with random hexamers. The cDNAs were quantified by real-time PCR using the FastStart SYBR Green Master (Roche), and the NP-specific primer set and GAPDH-specific primer set as follows: 5′-ACAGATGGGTGAAACAGC-3′ and 5′-CTCCAGAGCAATGGGTAG-3′ for CDV-NP; 5′-GTC AGCCGCATCTTCTTTTG-3′ and 5′-GCGCCCAATACGA CCAAATC-3′ for GAPDH. Real-time PCR was performed using a LightCycler 96 (Roche). Relative RNA levels compared with GAPDH and empty vector was calculated by the 2^–ΔΔ^ CT method.

### Co-immunoprecipitation assay

The effect of mAb on the interaction of H/F proteins was detected by Co-IP assay. The Flag-H and Myc-F plasmids of CDV 851 strain was respectively transfected into 293T cells with transfection reagent lipofectamine 2000 (Invitrogen, USA), and an empty vector was set up as the control. After 24 h of transfection, the cell samples were lysed in NP-40 buffer (Beyotime, Shanghai, China). Subsequently, equal aliquots of supernatants of Flag-H protein were immunoprecipitated with either mAb 2D12, or anti-Flag antibody at 4°C overnight, followed by incubation with protein A/G agarose beads (Santa Cruz, USA) for 4 h. After washing with NP-40 buffer, Myc-F protein of CDV 851 strain were added and incubated with the Flag-H material immunoprecipitated with either mAb 2D12 or anti-Flag antibody for 4 h. IP samples were obtained by washing with NP-40 buffer. The samples were then detected by western blot using anti-Flag and anti-Myc antibody. The effect of mAb on the interaction of H protein of CDV 851 strain and dogSLAM was detected as described above using anti-SLAM antibody and anti-Flag antibody.

### H/F-mediated membrane fusion

Vero cells in 24-well plates were co-transfected with 0.5 μg of pCA-F plasmid, 0.5 μg of pCA-H plasmid of CDV 851 strain with 3 μl of lipofectamine 2000 (Invitrogen, USA). 293T cells in 24 wells were transfected with 0.4 μg of pCA-H plasmid, 0.4 μg of pCA-F plasmid of CDV 851 strain, 0.2 μg of dogSLAM or without dogSLAM with 3 μl of lipofectamine 2000 (Invitrogen, USA). All transfections were performed according to the manufacturer’s protocol. After 6 h transfection, the supernatant was refreshed, and mAb 2D12 (final concentration of 2.5 μg/ml) was added. The views of cell-cell fusion were observed 48 h post-transfection with a microscope.

## Results

### Characterization of neutralizing mAb against CDV

**FIGURE 1:**
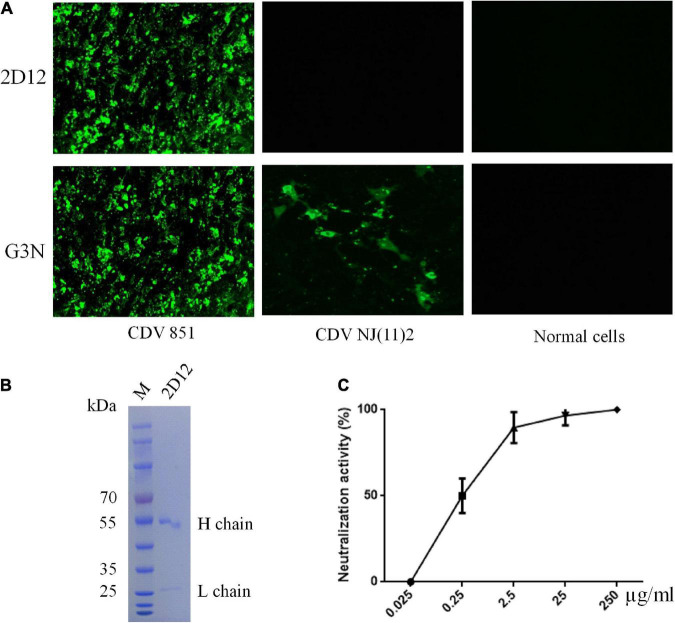
Characterization of mAb 2D12. **(A)** Identification of the reactivity of mAb 2D12 with CDV 851 strain in Vero and CDV NJ(11)2 in Vero-SLAM cells by immunofluorescence assay (IFA). The Vero cells were infected with CDV 851 strain, while Vero-SLAM cells were infected with CDV NJ(11)2 strain, which were inculated with mAbs 2D12 or G3N. **(B)** The purification of mAb 2D12. The mouse ascites of mAb 2D12 were purified by affinity chromatography using HiTrap TM Protein G and analyzed by SDS-PAGE. **(C)** Virus neutralization assay. The CDV 851 strain was incubated with the indicated dilution of purified mAb 2D12, the virus-antibody mixture was inoculated onto Vero cells, the cytopathic effect (CPE) was observed and the VN antibody titers expressed as the reciprocal of the highest plasma dilution giving 50% protection. Means ± S.D. of data from three independent experiments performed in triplicates are shown.

A mAb, designated as 2D12, was obtained using sp2/0 myelomas and splenocytes from mice immunized with purified CDV 851 strain as described under methods (Bi et al., 2015b). Reactivity and specificity of mAb was examined by immunofluorescence assay (IFA), the mAb 2D12 showed reactivity with Vero cells infected with CDV 851 strain but not Vero-SLAM infected with CDV NJ(11)2 and normal Vero cells, showing strain-specific reactivity, while the mAb G3N against CDV N protein (Bi et al., 2017) reacted with both strains of CDV 851 and CDV NJ(11)2 (Figure 1A). The mAb 2D12 was identified to be subclass IgG1κ and mice ascites was well purified by HiTrap Protein G Affinity chromatography column (Figure 1B). We measured the 50% inhibitory concentration (IC_50_) values of mAb 2D12 IgG using virus neutralization assays and found that the IC_50_ was 0.28 μg/ml (Figure 1C).

**FIGURE 2:**
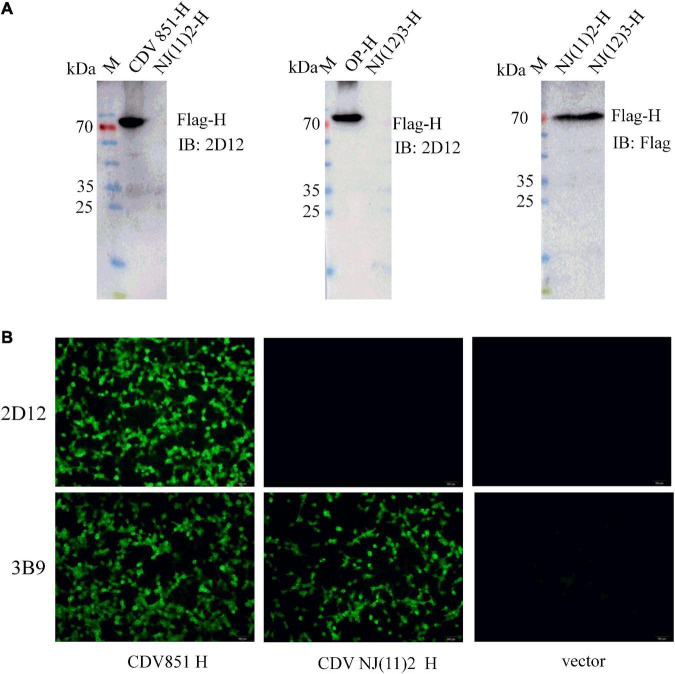
Identification of the reactivity of mAb 2D12 with H protein of different canine distemper virus (CDV) strains. **(A)** The reactivity of mAb 2D12 with H protein of different CDV strains by western blot. 293T cells were transfected with pCA-H plasmids of CDV 851, NJ(11)2, NJ(12)3, and OP strains, after 24 h post-transfection, the cell lysates were prepared and subjected to western blot analysis with mAb 2D12 and control Flag antibody. **(B)** The reactivity of mAb 2D12 with H protein of different CDV strains by immunofluorescence assay (IFA). 293T cells were transfected with pCA-H plasmids of CDV 851 and NJ(11)2 strains, after 24 h post-transfection, the cell lysates were fixed, followed by incubation with mAb 2D12, 2B9, and FITC-conjugated anti-mouse IgG antibody. The cells were observed under the fluorescence microscopy.

Considering that CDV H protein was the major neutralizing target (Hirayama et al., 1991; Bouche et al., 2002; de Swart et al., 2005, 2009), the reactivity of the mAb with H protein was examined. The recombinant plasmids of full-length H protein of different CDV strains were transfected into 293T cells. Reductive SDS-PAGE electrophoresis and western blot were performed to test the reactivity of mAb 2D12 with these H proteins. The results showed that mAb 2D12 reacted with the H protein of CDV 851 and Onderstepoort (OP) strains, but did not react with the H protein of NJ(11)2 strain of Asia-4 lineage and NJ(12)3 strain of Asia-1 lineage (Figure 2A), indicating that the linear antigenic epitope recognized by mAb 2D12 has been altered in H protein of CDV 851, OP strains and NJ(11)2, NJ(12)3 strains. IFA results showed that the mAb 2D12 reacted with H protein of CDV 851 strain, but did not react with H protein of CDV NJ(11)2 strain and empty vector (Figure 2B). These results indicated that mAb 2D12 had different reactivity with different CDV strains.

### Mapping of the epitope of H protein

**FIGURE 3:**
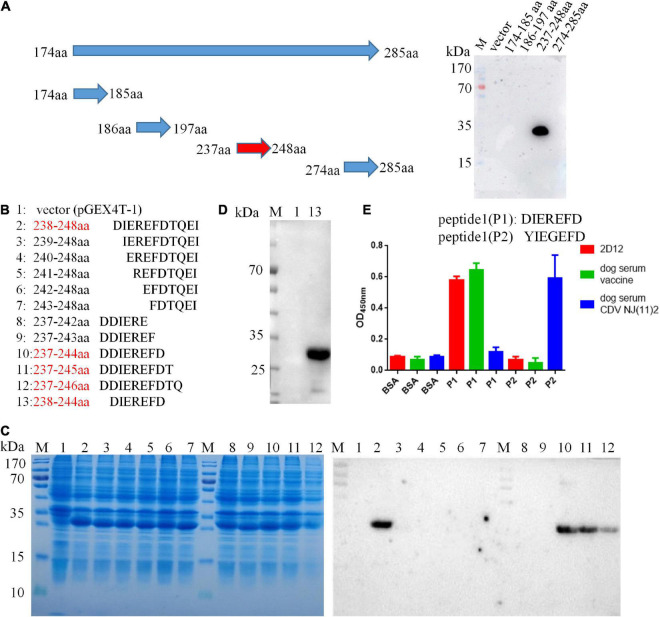
Epitope identification of mAb 2D12 by western blot. **(A)** Schematic representation of truncated H proteins of canine distemper virus (CDV) and identification of the reactivity of mAb 2D12 with truncated H fragments by western blot. **(B)** Amino acid sequences of different truncated proteins. The red text color indicated the positive reactivity of the truncated proteins with mAb 2D12. **(C)** The expression of different truncated H proteins was identified by SDS-PAGE and the reactivity of mAb 2D12 with different truncated H proteins was identified by western blot. **(D)** The reactivity of mAb 2D12 with the identified antigen epitope (238DIEREFD244) was identified by western blot. **(E)** Serological test for the identified epitope by ELISA. The reactivity of antigen epitope (238DIEREFD244 or 238YIEGEFD244) with mAb 2D12 and dog sera against vaccine or CDV NJ(11)2 strain was identified by ELISA. Means ± S.D. of data from three independent experiments performed are shown.

A panel of overlapping and truncated proteins were used to map the epitope. After screening, the neutralizing epitope recognized by 2D12 was defined in region aa 174-285 of H protein (Supplementary Figure 1). To further identify the precise epitope on the H protein of CDV, a panel of truncated proteins from H (aa 174-285) of CDV 851 strain were performed. After screening, the mAb 2D12 was reactive with the fragment (aa 237–248) (Figure 3A), indicating that the epitope was further defined within aa 237–248 of H protein. Subsequently, the reactive polypeptides were truncated by one residue at their C-terminus and/or N-terminus until the smallest binding domain recognized by the mAb was identified. The GST-fused fragments aa 238-248, aa 237-244, aa 237-245, and aa 237-246 were reactive with mAb 2D12, while the other polypeptides did not show the reactivity (Figures 3B, C). This result suggested that the polypeptide (238DIEREFD244) was the minimal linear epitope required for mAb 2D12 binding. Western blot further verified the epitope (238DIEREFD244) fused with GST tag could be recognized by mAb 2D12 (Figure 3D). The mAb 2D12 has different reactivity between CDV 851, OP strains and CDV NJ(11)2, NJ(12)3 strains used in this study and sequence alignment of H protein among the four strains showed the substitutions of two amino acid residues (D238Yand R241G) appeared in the epitope of CDV NJ(11)2, NJ(12)3, producing the epitope (238YIEGEFD244). To further test the effect of the two substitutions of amino acid residues on the reactivity of mAb 2D12, the two epitopes (238DIEREFD244) and (238YIEGEFD244) as antigens were synthesized and coupled to BSA to detect the reactivity of mAb 2D12 by ELISA. Compared with the epitope (238DIEREFD244), the mAb 2D12 did not recognize the epitope (238YIEGEFD244) with both substitutions (D238Y and R241G) (Figure 3E), which indicated that the two substitutions have a significant influence on the antigenicity of the epitope. Reactivity of the epitope with dog serum was also detected by ELISA with BSA-epitope as antigen. The results showed that the epitope (238DIEREFD244) reacted with mAb 2D12 and serum of dog immunized with vaccine (Onderstepoort), but not serum of dog infected with CDV NJ(11)2 strain. While the epitope (238YIEGEFD244) could be recognized by positive dog serum against CDV NJ(11)2 strain instead of vaccine Onderstepoort. According to the data of Figure 3E, we could draw the conclusion that the identified epitope is targeted to dog immune response during the virus infection, which resulting in strain-specific immune response.

### Alignment analysis of the defined epitope

**FIGURE 4:**
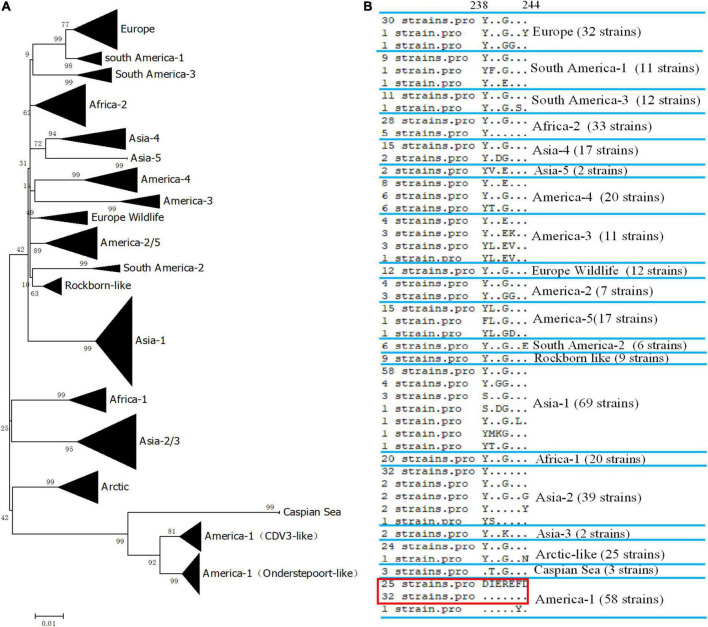
Conservative analysis of antigen epitope. **(A)** Phylogenetic relationships based on the H genes of 405 canine distemper virus (CDV) strains. Distance values were calculated by the Clustal W program using the MEGA 6.0 software package. The tree was constructed by the Maximum-Likelihood method, and bootstrap values were calculated on 1,000 replicates. **(B)** Alignment of amino acids sequence of the neutralizing epitope in different lineages of CDV strains. The dots indicated the same amino acids with the epitope 238DIEREFD244 in red box.

We did not have enough strains to test the reactivity of the mAb 2D12. Therefore, we evaluated the reactivity of mAb 2D12 with different strains by comparing conservation and variability of the neutralizing epitope sequence. The H protein sequences of 405 representative CDV strains of different lineages were selected from GenBank (Supplementary Table 1) for comparison. The epitope 238DIEREFD244 of CDV 851 strain was conserved in 25 CDV strains belonging to CDV3 lineage, was also conserved in 32 CDV strains of Onderstepoort (except GZ0804 strain), which both belonged to America-1 vaccine lineage (Figure 4A). The result showed that the neutralizing epitope recognized by mAb 2D12 was highly conserved among America-1 vaccine strains and the mAb 2D12 could recognize these strains. Different from CDV 851 and OP strains, CDV NJ(11)2 and CDV(12)3 strains had D238Y and R241G mutations, forming a variant neutralizing epitope (238YIEGEFD244). Further, alignment with sequences from the databases showed that all CDV strains of different lineages has different substitutions of amino acid residues in the epitope (Figure 4B and Supplementary Table 1). These suggested that the epitope recognized by 2D12 was variable and might be used for differentiation between America-1 lineage vaccine and the other lineages of CDV strains. In conclusion, neutralizing epitope (238DIEREFD244) is lineage-specific epitope of America-1 vaccine lineage and the other lineages of CDV strains, thus the mAb 2D12 could also distinguish the CDV strains between America-1 vaccine lineage and the other lineages.

### Neutralizing mAb does not inhibit receptor binding

**FIGURE 5:**
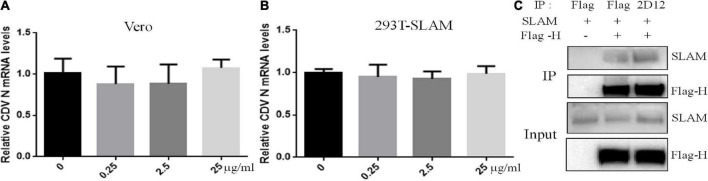
Effect of mAb 2D12 on adsorption of canine distemper virus (CDV) to cells and the interaction between H protein and dogSLAM receptor. **(A)** The effect of mAb 2D12 on adsorption of CDV 851 to Vero cells was detect by RT-qPCR. CDV 851 and mAb 2D12 were mixed and inoculated into monolayer Vero cells. The N mRNA level of CDV adsorbing on the Vero cells was detected by RT-qPCR. **(B)** The effect of mAb 2D12 on adsorption of CDV 851 to 293T/SLAM cells was detect by RT-qPCR. CDV 851 and mAb 2D12 were mixed and inoculated into monolayer 293T/SLAM cells. The N mRNA level of CDV adsorbing on the 293T/dogSLAM cells was detected by RT-qPCR. Means ± S.D. of data from three independent experiments performed are shown. **(C)** The effect of mAb 2D12 on the interaction between Flag-H protein CDV 851 strain and dogSLAM by Co-IP assay. The Flag-H and dogSLAM proteins respectively expressed in 293T cells were immunoprecipitated with either mAb 2D12 or anti-Flag antibody, the precipitated Flag-H material was labeled with an anti-Flag antibody, and the coprecipitated dogSLAM protein was detected with an anti-SLAM antibody.

To mechanistically explore the basis for neutralization by mAb 2D12, the effect of mAb 2D12 on virus adsorption to cells were performed by virus adsorption experiment. The amount of virus adsorbed on Vero cells was detected by RT-qPCR. The result showed that mAb 2D12 could not inhibit the adsorption of CDV 851 strain on Vero cells (Figure 5A). CDV 851 strain could not infect 293T cells, but could infect 293T cells expressing dog SLAM receptor (Data not shown). Virus binding test was carried out on 293T/dogSLAM cells. RT-qPCR showed that mAb 2D12 could not inhibit the adsorption of CDV 851 strain to 293T/dogSLAM cells (Figure 5B), indicating that mAb 2D12 could not inhibit the binding of virus to dogSLAM receptor. Further, the effect of mAb 2D12 on the interaction between H protein and dogSLAM receptor was analyzed by Co-IP. The result showed that compared with Flag antibody, the binding of mAb 2D12 to H protein did not affect the binding of H protein to dogSLAM receptor, further indicating that mAb 2D12 did not block the binding of H protein to dogSLAM receptor (Figure 5C).

### Syncytium formation of H/F is blocked by mAb 2D12

**FIGURE 6:**
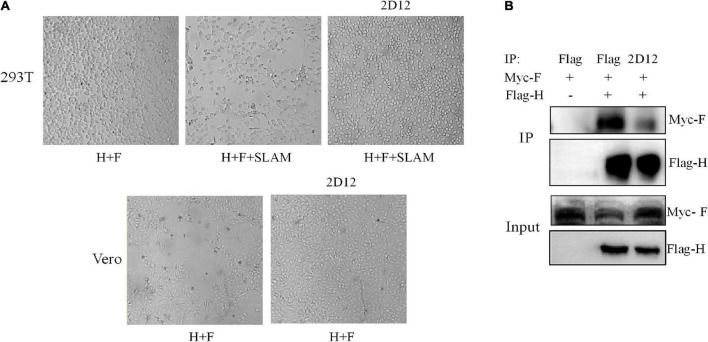
Effect of mAb 2D12 on membrane fusion and interaction between H and F proteins. **(A)** Inhibition of H/F-mediated membrane fusion by mAb 2D12. Cell-to-cell fusion activity in Vero cells or 293T-dogSLAM cells triggered by co-expression of H and F of CDV 851 strain, in the presence (+) of absence (–) of mAb 2D12. Representative fields of view of cell-cell fusion induced 48 h post-transfection are shown. **(B)** Identification of the effect of mAb 2D12 on the interaction between H and F proteins by Co-IP assay. The Flag-H and Myc-F proteins of CDV 851 strain respectively expressed in 293T cells were immunoprecipitated with either mAb 2D12 or anti-Flag antibody, the precipitated Flag-H material was labeled with an anti-Flag antibody, and the coprecipitated Myc-F protein was detected with an anti-Myc antibody.

In order to determine whether the fusion capacity of H/F was blocked by mAb 2D12, we expressed H and F proteins of CDV 851 strain in 293T and Vero cells. The co-expression of H/F was not able to induce the formation of multinucleated syncytia in 293T cells (Figure 6A). Interestingly, syncytium formation was detectable following H/F and SLAM expression in 293T cells. This finding was in agreement with results that the cell fusion induced by H/F was dependent on receptors (Avila et al., 2014). Following transfection of 293T/SLAM cells with the H/F construct, the cells were incubated with 2.5 μg/ml of mAb 2D12, this result showed that mAb 2D12 directed against H protein could inhibit H/F-mediated fusion (Figure 6A). Expression of H and F proteins in Vero cells could induce to form polynuclear syncytia, and this fusion activity was also inhibited by mAb 2D12 (Figure 6A). It can be speculated that the epitope recognized by mAb 2D12 may be involved in the fusion steps other than the receptor binding.

### The neutralizing mAb interferes with interaction of H-F protein

The formation of a stable complex between H and F protein is the premise of membrane fusion. To examine whether the H-F protein interaction was affected by mAb 2D12, co-immunoprecipitation of CDV envelope glycoprotein complexes was performed. The results showed that compared with Flag antibody, when monoclonal antibody 2D12 binds to H protein, the amount of F protein immunoprecipitated with H protein was significantly reduced, indicating that the neutralizing mAb 2D12 interferes with the interaction between H and F protein for inhibiting membrane fusion (Figure 6B).

### Spatial location of the epitope

**FIGURE 7:**
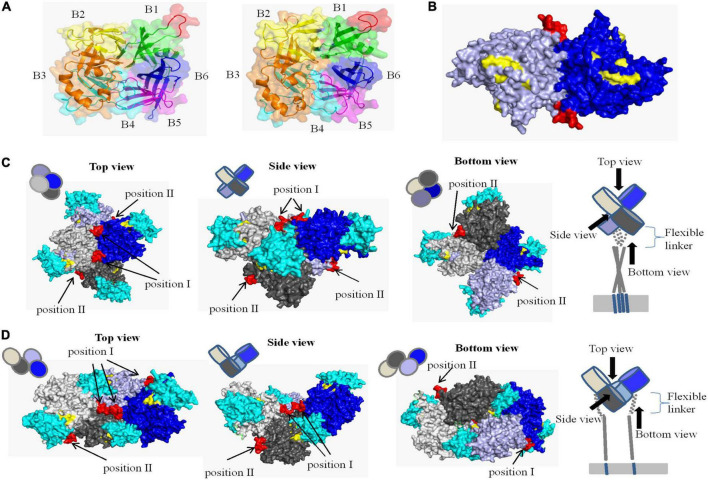
Structural location analysis of the neutralizing epitope on the CDV 851 H protein. **(A)** Localization of the neutralizing epitope on monomer of CDV 851 strain (left) and CDV NJ(11)2 strain (right). The amino acid residues on sheets 1, 2, 3, 4, 5, and 6 are shown in green, yellow, orange, cyan, purple, and blue, respectively. **(B)** Localization of the neutralizing epitope on dimers. The two H protein molecules are shown in blue and light blue. **(C,D)** Localization of the neutralizing epitope on tetramer in form I **(C)** (Tahara et al., 2013) and form II **(D)** (Tahara et al., 2013). The four H protein molecules are shown in gray, light gray, blue, and light blue and dogSLAM is shown in cyan. The epitope recognized by mAb 2D12 is shown in red. The residues on the surface of canine distemper virus (CDV) H protein implicated in SLAM binding are highlighted yellow.

The crystal structure of CDV H protein is not available, but the crystal structure of measles virus (MV) H protein, which belongs to the same genus as CDV, has been resolved and represents the norm for hemagglutinins of *Morbillivirus spp* (Colf et al., 2007). Therefore, we were able to localize the identified epitope on a predicted 3D structure. On the SWISS-MODEL website, the crystal structures of MV H protein 2rkc (monomer), 2zb6 (dimer), 3alw (tetramer form I), and 3alx (tetramer form II) were used as template to homology modeling H protein of CDV-L strain, and PyMOL software was used to determine the position of neutralizing epitope on the structure of H protein. The homology of H protein of CDV 851 and CDV NJ(11)2 strains were 34.4 and 36.1% with MV, which is more than 30% (Supplementary Figure 2). Similar to MV (Colf et al., 2007; Hashiguchi et al., 2007, 2011), the overall fold of CDV H was that of a β-propeller with six blades. Each of the blade modules, B1–B6, contained four antiparallel β-strands, S1–S4. The blades were connected sequentially through extended loops between S4 of one module and S1 of the next. The identified epitope (red) was located the loop between S2 and S3 of B1 and was completely exposed to the surface of H protein (Figure 7A), so it may be a major neutralizing target site to stimulate easily produce antibody. In addition, we observed different conformational structures including the identified epitope in CDV 851 strain (Figure 7A left) and CDV NJ(11) 2 strain (Figure 7A right), suggesting the mutations changed conformational structure of the H protein even antigen. In the dimer structure, the neutralizing epitope was located at the contact surface of the two H monomers (Figure 7B). The higher order (tetrameric) structures of the CDV H protein-SLAM complex were proposed on basis of the crystal structures of MV. There are two models for the tetramer structure of CDV H protein. The neutralizing epitope had two localizations in both models of tetramer. The neutralizing epitope of position I formed part of the interface of H protein dimer, while the neutralizing epitope of position II was exposed on the lateral surface of monomer H protein head (Figures 7C, D). The neutralizing epitopes of position I and position II were not located at the position of RBS (Figures 7C, D), indicating that this neutralizing epitope had nothing to do with the binding of H protein to the receptor. These data were consistent with the observation that mAb 2D12 recognizing the epitope did not inhibit the binding of virus to cells (Figure 5).

## Discussion

At present, the control of CDV still remains a serious challenge to veterinarians. CDV H envelope glycoprotein represents an attractive target for vaccination or passive immunotherapy. But H protein has high mutations, resulting in distinct CDV lineages known as America 1-5, Asia 1-5, South America 1-3, Europe, Europe Wildlife, Arctic, Rockborn-like, Africa 1-2, and Caspian Sea (Espinal et al., 2014; Bi et al., 2015a; Anis et al., 2018a; Duque-Valencia et al., 2019; Rendon-Marin et al., 2019; Wang et al., 2022). Except for lineage diversity, the antigenic difference among CDV strains have been reported (Anis et al., 2018b). We have previously reported antigenic variation in two different neutralizing conformational epitopes of H protein (Bi et al., 2015b). Current vaccines may provide incomplete protection due to antigenic difference, so it’s necessary to clarify the detailed neutralizing epitopes of CDV H protein. Shi et al. (2020) identified two linear epitopes 120QKTNFFNPNREFDFR134 and 178ARGDIFPPY186 of H protein of CDV, but both of them were not neutralizing epitopes. Only a linear neutralizing epitope has previously been identified to be aa 126–133 of CDV H protein (Ader-Ebert et al., 2015). In this study, we generated a neutralizing mAb 2D12 against CDV 851 strain, and it binding epitope was mapped to a linear epitope 238DIEREFD244. The epitope was highly conserved in America-1 vaccine lineage strains, while different mutations occured in all CDV strains of the other lineages in this study. The CDV strains of Caspian Sea which were wild-type strains most closely related to American-1 vaccine strains also had two mutations in this neutralizing epitope (DTEGEFD), at positions 2 and 4, thus providing additional evidence that this epitope may be of some significance. The Rockborn vaccine strain had been withdrawn from several markets, because it was less attenuated and less safe than other CDV vaccines, e.g., Onderstepoort strain (Martella et al., 2010). Interestingly, Rockborn vaccine strains of CDV also differed by two amino acids in this epitope (YIEGEFD), indicating that the variation in this epitope is most likely related to lineage specific differences. We revealed that D238Y and R241G mutations in most strains of some lineages eliminated the reactivity of mAb 2D12. The antigenic change of the epitope also resulted in strain-specific immune response in dogs. This is the first time to verify the antigenic difference between America-1 vaccine and wild-type strains of CDV at the epitope level. These results suggest that mAb 2D12 and its targeting epitope may represent a valuable diagnostic tool for distinguishing between America-1 vaccine and the other lineages of CDV strains.

The structural model demonstrated that the linear epitope recognized by mAb 2D12 was shown to be fully exposed on the surface of the H protein, and located at the loop between S2 and S3 of B1, which was so flexible that little electron density was observed in the H crystal structures (Hashiguchi et al., 2011). These support that the epitope has good antigenicity. The flexible region may make the epitope prone to structural change. We observed that the epitope in CDV 851 and NJ(11)2 strains showed the different structure, suggesting the mutations changed conformational structure of the antigen. However, the different conformational structures may not be related to the change of reactivity of the epitope with mAb 2D12, because the epitope was identified as a linear epitope. In addition to the identified epitope, different conformational structures were also observed due to amino acid mutations, this may contribute to the change of antigenicity, as we have previously observed antigenic difference of the conformational epitopes in H protein (Bi et al., 2015b).

At the initial stage of CDV infection, the H protein binds to different cellular receptors and then triggers membrane fusion (Ader-Ebert et al., 2015; Plattet et al., 2016). The two processes can be targeted for suppression to implement antiviral strategies (Bovier et al., 2021; Shrestha et al., 2021). Ader-Ebert et al. (2015) report that an anti-CDV H mAb targeting the linear H-stalk segment aa 126–133, potently abrogated the F-triggering function of H-protein for inhibiting membrane fusion without impairing binding of H protein to receptors or H-F interaction. The residues (501D, 503D, 521V, 525Y, 526D, 527I, 529R, 532H, 540D, 542I, 549Y, and 550P) of CDV H protein were implicated in receptor binding as reported in the literature (Colf et al., 2007). In this study, we found that a mAb 2D12 neutralized CDV infection by inhibiting virus fusion without affecting the receptor binding, this was also supported by the result that neutralizing epitope recognized by mAb12 was not located at the position of RBS (Figures 7B–D). These data suggested that mAb12 was involved in membrane fusion of virus, rather than the receptor binding.

Membrane fusion of MV is mediated by concerted actions of the H and F proteins, which was called membrane fusion apparatus (Navaratnarajah et al., 2016). In this study, the mAb 2D12 reduced the interaction of H protein and F protein for neutralizing virus, suggesting that mAb 2D12 was involved in the stability of membrane fusion apparatus. No structural data of F protein in complex with the attachment H protein is currently available, thus precluding exact understanding of the epitope in H-F mode of interactions. The morbilliviruses H and F glycoproteins are thought to associate already intracellularly prior to receptor binding (Bose et al., 2015). The H-stalk region aa 110–118 was identified as a putative candidate region involved in short-range interaction with the Ig-like microdomain of the F globular head domain (Lee et al., 2008; Plattet et al., 2016). The epitope (aa 238–244) in this study was not overlapped with the H-stalk region aa 110–118, thus the mAb 2D12 did not inhibit the functional cross-talk between the H and F proteins in a direct way. The mAb 2D12 may exert some steric hindrance to reduce the contact of the H-stalk central region with F protein. The epitope (position I) formed part of the interface of the H protein dimers. The mAb 2D12 targeting the epitope (position I) may affect proper tetramerization of the H protein for inhibiting the interaction of H and F protein. We could not exclude the effect of mAb 2D12 on the conformational or topographical rearrangements of H protein, which is a key for H-F protein oligomeric structure to trigger fusion (Navaratnarajah et al., 2016; Plattet et al., 2016).

In summary, we generated a novel neutralizing mAb 2D12 against CDV H protein, and identified a lineage-specific neutralizing epitope for differentiation of America-1 vaccine lineage and the other lineages of CDV strains. The epitope had two different positions in the tetramer structure of CDV H protein, mAb 2D12 targeting the epitope inhibited the formation of H and F protein complex for neutralizing virus. Our study will enrich understanding of the antigenic epitopes present in CDV H protein and the mechanism of action of H protein in membrane fusion. The study may also be useful for further developing the diagnostic tools and new antiviral strategies for CDV infections.

## Data availability statement

The datasets presented in this study can be found in online repositories. The names of the repository/repositories and accession number(s) can be found in the article/Supplementary material.

## Ethics statement

The studies involving animals were reviewed and approved by the Committee on the Ethics of Animal Experiments of Jiangsu Academy of Agricultural Sciences, with the approval number SYXK (Su) 2020-0024.

## Author contributions

ZB performed and designed the experiments, supervised the study, and drafted the manuscript. WW and XX helped to analyze the data. All authors read and approved the final manuscript.

## Supplementary material

Supplementary Figure 1 Identification of the reactivity of mAb 2D12 with different truncated H proteins. The expression of different truncated H proteins of CDV 851 strain was identified by SDS-PAGE and the reactivity of mAb 2D12 with different truncated H proteins was identified by western blot. The positive reaction of the truncations with mAb 2D12 was marked by red.

Supplementary Figure 2 Alignment analysis of H protein of CDV 851, CDV NJ(11)2 strains and MV strain. The same amino acids are shown, the dot (.) indicated different amino acids from MV strain.

Supplementary Table 1 Information of different lineages of CDV isolates used in Figure 4.
