# Supplementary figures and images for: Structure and function of a novel lineage-specific neutralizing epitope on H protein of canine distemper virus
Source: Front Microbiol. 2023 Jan 11;13:1088243. doi: 10.3389/fmicb.2022.1088243 (PMC9875009; doi:10.3389/fmicb.2022.1088243)

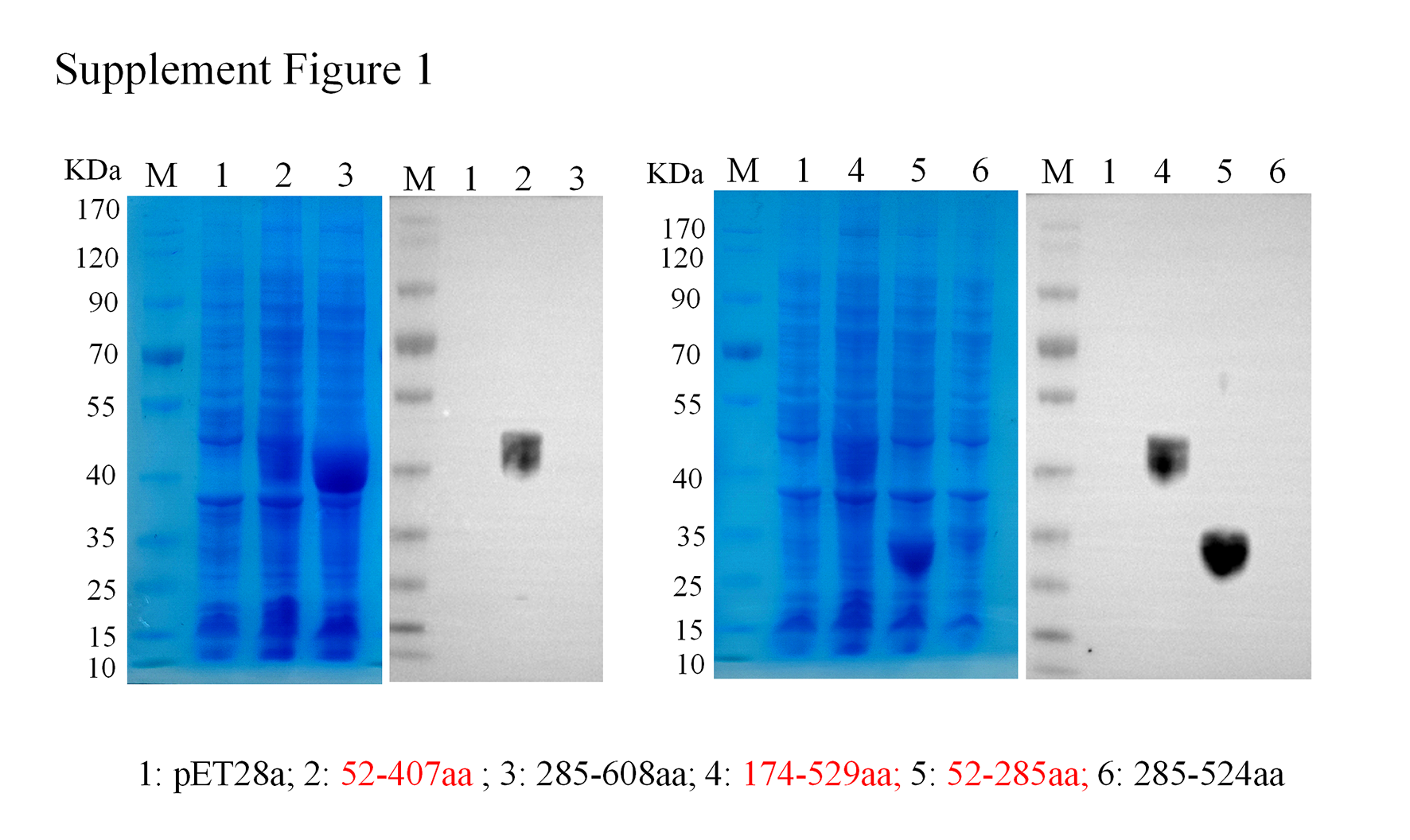

Supplement: Supplementary Figure 1 — Identification of the reactivity of mAb 2D12 with different truncated H proteins. The expression of different truncated H proteins of CDV 851 strain was identified by SDS-PAGE and the reactivity of mAb 2D12 with different truncated H proteins was identified by western blot. The positive reaction of the truncations with mAb 2D12 was marked by red. [file Image_1.TIF]

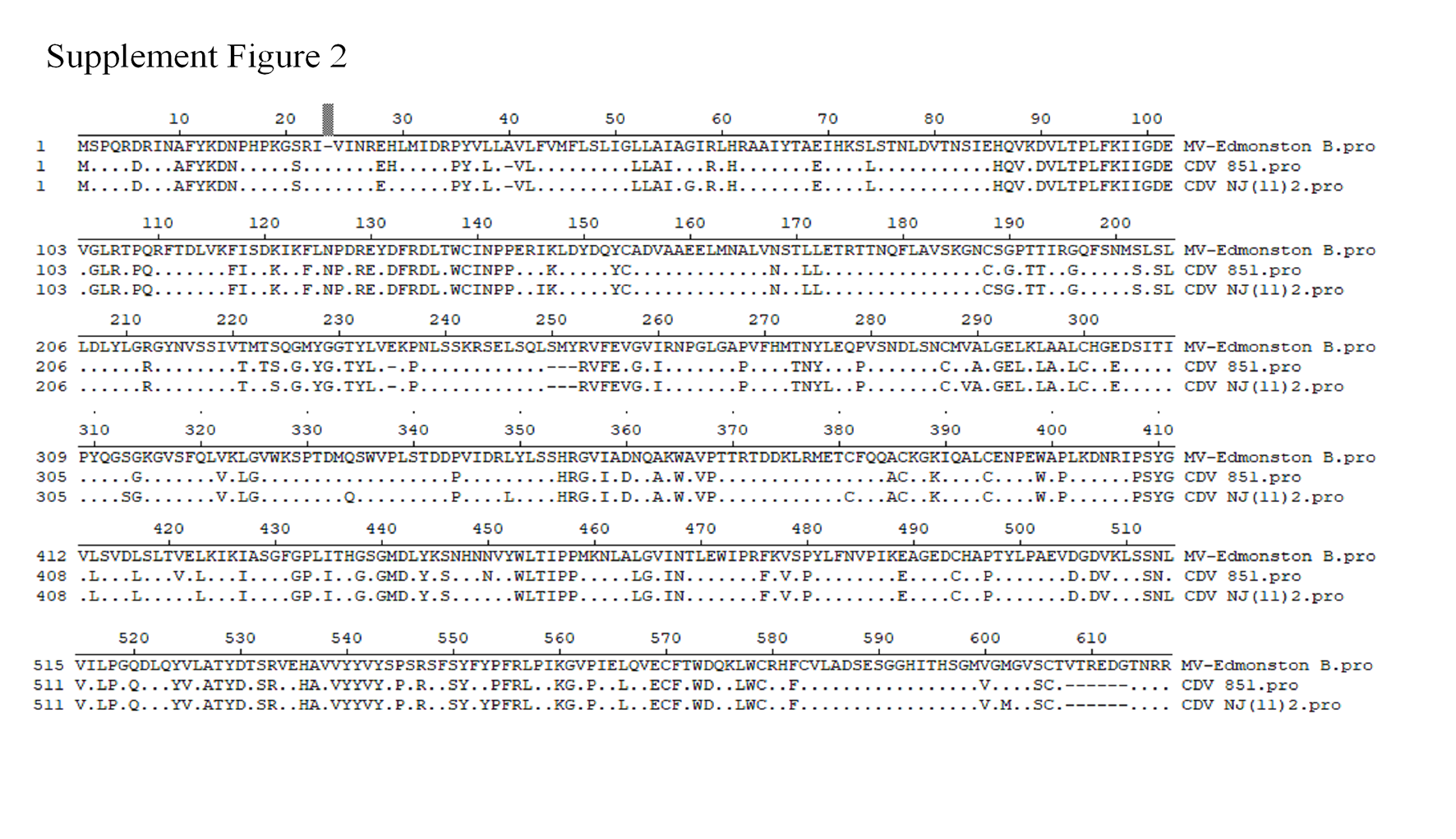

Supplement: Supplementary Figure 2 — Alignment analysis of H protein of CDV 851, CDV NJ(11)2 strains and MV strain. The same amino acids are shown, the dot (.) indicated different amino acids from MV strain. [file Image_2.TIF]
